# Polymeric Nanoparticles’ Accumulation in Atopic Dermatitis: Clinical Comparison between Healthy, Non-Lesional, and Lesional Skin

**DOI:** 10.3390/pharmaceutics15071927

**Published:** 2023-07-11

**Authors:** Céline Try, Mona M. A. Abdel-Mottaleb, Arnaud Béduneau, Brice Moulari, Lionel Pazart, Chrystelle Vidal, Gaëlle Brunotte, Florence Castelain, Alf Lamprecht, Philippe Humbert, Yann Pellequer

**Affiliations:** 1 PEPITE EA4267, (Labex LipStic ANR-11-LABX0021) Université Franche-Comté, F-25000 Besançon, France; celine.try@univ-fcomte.fr (C.T.); arnaud.beduneau@univ-fcomte.fr (A.B.); brice.moulari@univ-fcomte.fr (B.M.); lamprech@uni-bonn.de (A.L.); 2 CHU de Besançon, F-25000 Besançon, France; lpazart@chu-besancon.fr (L.P.); cvidal@chu-besancon.fr (C.V.); gbrunotte@chu-besancon.fr (G.B.); fcastelain@chu-besancon.fr (F.C.); 3 Department of Pharmaceutics and Industrial Pharmacy, Faculty of Pharmacy, Ain Shams University, Cairo 11566, Egypt; 4 INSERM CIC 1431, CHU de Besançon, F-25000 Besançon, France; 5 Department of Dermatology, Allergology Unit, CHU de Besançon, F-25000 Besançon, France; 6 Department of Pharmaceutics, Institute of Pharmacy, University of Bonn, 53121 Bonn, Germany; 7 RIGHT UMR1098 INSERM EFS BFC, Université Franche-Comté, F-25000 Besançon, France; philippe.humbert@univ-fcomte.fr

**Keywords:** atopic dermatitis, polymeric nanoparticles, tight junction, clinical study, altered permeability

## Abstract

A major limitation in the current topical treatment strategies for inflammatory skin disorders is the inability to selectively target the inflamed site with minimal exposure of healthy skin. Atopic dermatitis is one of the most prevalent types of dermatitis. The use of polymeric nanoparticles for targeting inflamed skin has been recently proposed, and therefore the aim of this proof-of-concept clinical study was to investigate the skin penetration and deposition of polymeric biodegradable nanoparticles in the atopic dermatitis lesions and compare the data obtained to the deposition of the particles into the healthy skin or lesion-free skin of the atopic dermatitis patients. For that, fluorescent PLGA nanoparticles in sizes of approximately 100 nm were prepared and applied to the skin of healthy volunteers and the lesional and non-lesional skin of atopic dermatitis patients. Skin biopsies were examined using confocal laser scanning microscopy to track the skin deposition and depth of penetration of the particles. Immunohistochemistry was performed to investigate the alteration in tight-junction protein distribution in the different types of skin. Results have shown that nanoparticles were found to have higher deposition into the atopic dermatitis lesions with minimal accumulation in healthy or non-lesional skin. This has been primarily correlated with the impaired barrier properties of atopic dermatitis lesions with the reduced production of Claudin-1. It was concluded that polymeric nanoparticles offer a potential tool for selective drug delivery to inflamed skin with minimal exposure risk to healthy skin.

## 1. Introduction

Atopic dermatitis (AD), also known as atopic eczema, is a chronic, relapsing inflammatory skin disorder characterized by a major impairment in the stratum corneum barrier properties resulting in disturbed skin function, dehydration, and the release of a series of inflammatory cytokines [1]. At the clinical level, atopic dermatitis patients experience skin dryness, itching, erythema, scaling, and fissuring [2]. The onset of the disease occurs anytime during one’s life, with an increased tendency of patients to produce immunoglobulin E (IgE) antibodies if exposed to environmental or ingested allergens [3]. The immunological response leads to severe pruritus initially but could also progress to more severe inflammatory conditions that may affect other body organs, primarily the respiratory system and the eye, in what is known as the atopic march [4]. With chronic inflammation, impaired epidermal progenitor cells’ proliferation usually leads to epidermal hyperplasia and spongiosis of the skin, leaving it highly susceptible to different types of infections. Since the prevalence of the disease is high in children, the disease significantly affects their quality of life, with frequent skin scratching and the associated sleep disturbance necessitating high care, similar to caring for type I diabetic children [5]. The first-line treatment of AD includes non-pharmacologic interventions related primarily to the use of emollients and different moisturizing modalities to increase skin hydration and lessen the pruritus, erythema, and fissuring in addition to frequent bathing to remove the crust, irritants, and scales from the skin surface. The pharmacologic treatment of symptoms is usually performed by the use of topical corticosteroids to ameliorate the active inflammatory phase and reduce relapses. The chronic nature of the disease necessitates the long-term concomitant use of corticosteroids and moisturizing agents, which enhance their systemic absorption with subsequent systemic side effects in addition to the commonly known topical side effects such as skin atrophy, rosacea-like eruptions, telangiectasia, and skin purpura, which usually leads to secondary skin infections [6]. Therefore, the use of targeted drug delivery to enhance the concentration of drugs on inflamed skin parts and minimize the exposure of healthy skin would offer a great advantage during the treatment of AD [7]. This could be correlated with the possible disturbance of skin barrier properties in the case of diseases such as dermatitis, which can be used to enhance the targeting potential of certain drug-delivery systems. It is known that the outermost layer of the skin, “the stratum corneum” with the high lipid content of ceramides, cholesterol, and fatty acids in excellent packing, represents the first barrier layer against xenobiotic skin permeation. Additionally, tight junctions play a crucial role in the skin barrier properties, especially in hair follicles and skin appendages lacking the stratum corneum. They are composed of various types of transmembrane proteins acting synergistically to regulate water and solute permeation through the skin. Recently, the compromised barrier properties of inflamed skin have led to the enhanced accumulation of certain nanosystems in diseased areas of the skin. Polymeric nanoparticles have offered a great tool to enhance the localization of topical drugs in diseased skin in addition to their ability to provide higher stability and the controlled release of different hydrophilic and lipophilic drugs [8,9,10]. Selective preferential accumulation of ethyl cellulose nanoparticles in the size range of 50–100 nm in dithranol-induced dermatitis on mice skin was observed while minimal accumulation was obtained on healthy skin [11], an observation that was even more pronounced upon using charged particles [12]. The encapsulation of betamethasone into 100 nm particles led to a significant improvement in therapeutic efficiency in irritant dermatitis due to the enhanced accumulation in the inflamed skin, apparently by a mechanism similar to the enhanced permeation and retention observed in tumors. The use of Poly L-lactide-co-glycolide acid (PLGA) as a biodegradable polymer would offer an additional advantage regarding the biocompatibility of the particles used. The polymer can simply biodegrade into safe monomers of lactic acid and glycolic acid and has been approved by the United States FDA and the European Medicine Agency in drug-delivery systems for parenteral applications. The penetration of 70 nm and 300 nm fluorescent PLGA nanoparticles into oxazolone-induced AD in mice ears revealed that there was no skin penetration of any of the particles into the healthy skin while the accumulation of the smaller particles was 15-fold higher than the larger particles into the inflamed skin areas. The same observation was obtained when using the same particles but on a porcine model of AD, a model considered to be the most similar to human skin [13]. This underlines the potential of smaller nanoparticles as a promising carrier for the treatment of AD. Therefore, the main objective of this study was to evaluate the penetration of polymeric nanoparticles in a size range of 100 nm in human atopic dermatitis. For that purpose, fluorescent PLGA particles were applied to the skin of healthy volunteers and atopic dermatitis patients. Additionally, a comparison between the penetration into atopic dermatitis lesions and non-affected skin areas in the patients was performed. Confocal laser scanning microscopy was used to investigate the deposition of the particles in skin biopsies of all different conditions. 

## 2. Materials and Methods

### 2.1. Materials

Poly (L-lactide-co-glycolide acid) (PLGA 50:50) Resomer^®^ RG502H Mwt 7000–17,000 was obtained from Evonik AG (Ingelheim, Germany). Fluorescein sodium salt, Polyvinyl Alcohol (PVA), ethyl acetate, Bovine serum albumin (BSA), Tween 80, and 4’, 6-diamidino-2-phenylindole (DAPI) were purchased from Sigma–Aldrich (Saint-Quentin Fallavier, France). The Anti-ZO1 Rabbit polyclonal tight junction protein antibody (ab216880), Goat Anti-Rabbit IgG H&L (Alexa Fluor^®^ 488, ab150077), Alexa Fluor^®^ 488. Ex: 495 nm, Em: 519 nm were obtained from Abcam France. The Anti-claudin-1 Antibody (A-9): sc-166338 –PE and Anti-claudin-4 Antibody (A-12): sc-376643 were purchased from Santa Cruz Biotechnology. All other chemicals were of analytical grade or equivalent quality. 

### 2.2. Methods

#### 2.2.1. Preparation of the Fluorescent PLGA Nanoparticles

The fluorescent PLGA nanoparticles were prepared using the double emulsion solvent evaporation technique. Briefly, fluorescein sodium was dissolved in ultrapure water at a concentration of 2.5 mg/mL. Next, 200 µL of this solution was then added to the PLGA solution in ethyl acetate, and the solutions were mixed using magnetic stirring for 15 min to obtain the primary emulsion. The obtained emulsion was then added to 10 mL of 0.75% PVA solution and homogenized using an ultrasonic probe (Sonopuls Bandelin, Berlin, Germany) for 4 min at the power of 100 watts. The organic solvent was then evaporated using a rotary evaporator (Büchi Rotavapor RE 120). The obtained nanoparticles were then washed using ultrapure water for 3 cycles of centrifugation at 14,000 rpm (equivalent to 20,817× *g*) for 45 min at 4 °C [14]. The washed particles were mixed with saccharose at a concentration of 1% ***w/v*** and kept at −20 °C for one night before being lyophilized for 24 h at −50 °C. 

#### 2.2.2. Characterization of the Nanoparticles

The particle size and the particle size distribution of the prepared nanoparticles were analyzed using dynamic laser scattering (Malvern Zetasizer Nano, Malvern Instruments Ltd., England, UK) [15]. Samples were dispersed in distilled water and analyzed at 25 °C. Zeta potential was determined using the same equipment [16]. For the determination of the entrapment efficiency of the fluorescein, the lyophilized nanoparticles were dissolved in dichloromethane, and the fluorescence intensity was evaluated using a microplate reader. All samples were analyzed in triplicates. 

For clinical use of the nanoparticles, a residual solvent assay and a bacterial load evaluation were necessary. Ethyl acetate is classified as one of the class 3 solvents, which have a maximum limit of 5000 ppm as a residual solvent. This concentration corresponds to a 50 mg/day maximum exposure dose. The determination of the residual solvent in the nanoparticles was performed using Gas chromatography according to the European Pharmacopeia 8.0 (monograph 5.4). In addition, the microbial count was evaluated according to the European Pharmacopeia 8.0 (monograph 5.1.4). For a topical formulation, the total aerobic microbial count must be less than 10^2^ CFU/mL and the total mold and yeast count should be less than 10^1^ CFU/mL. No Staphylococcus aureus or Pseudomonas aeruginosa should be detected in the clinically used formulation. 

#### 2.2.3. Clinical Study

##### Selection Criteria of Volunteers and Patients

Healthy volunteers and patients were recruited by the dermatology department of the Besançon University Hospital (CHRU de Besançon, France). To be included, volunteers and patients had to be Caucasian males under 65 years of age or females of childbearing age with a negative pregnancy test dated less than 3 weeks. Healthy volunteers had to have no history of skin conditions such as psoriasis or eczema. Patients had to have atopic dermatitis lesions larger than 1.5 cm^2^ on the anterior aspect of the forearm. A washout of topical treatments (class I or II dermocorticoids) on the forearms was performed two weeks prior to patient inclusion. The exclusion criteria of patients or volunteers included patients or volunteers who were healthy, underage, pregnant or breastfeeding, treated with topical or oral medications affecting skin penetration, had a coagulation deficiency, were unable to follow the protocol, did not sign the informed consent form, or were allergic to fluorescein sodium or other components of the formulation. This study obtained the agreement of the Ethics Committee of CHRU Besançon (identification number 13/692; approval date: 15 December 2014) and is registered by Agence Nationale de Sécurité du Médicament et des Produits de Santé (N° EudraCT: 2013-000318-39). The study is conducted according to the ethical principles of the Declaration of Helsinki. The patients and healthy volunteers participating in this study gave written consent. Ten healthy volunteers and six atopic dermatitis patients were selected for the study as summarized in Table 1. 

##### Application of the Fluorescent Nanoparticles

For healthy volunteers, nanoparticles were dispersed in distilled water and 800 µL of fluorescent nanoparticles were applied to a 10 cm × 4 cm area without massage on one of the forearms. For patients, 800 µL of particles was applied on an area of the same size containing atopic dermatitis lesions on the forearm. The same volume was also applied to another area of the forearm without lesions. Three days after topical application, one 3 mm × 3 mm punch for healthy volunteers and two punches for patients were made on both the lesional and non-lesional application areas.

##### Examination of the Skin Using Confocal Laser Scanning Microscopy (CLSM)

To follow the penetration of polymeric nanoparticles into different skin layers of the healthy volunteers and AD patients, skin biopsies were examined directly by confocal laser scanning microscopy (Olympus FV1000) using the ×10 objective. The system was equipped with an argon laser (excitation wavelength 488 nm) and a HeNe laser (excitation wavelength 568 nm) to observe fluorescence from nanoparticles (green) and skin autofluorescence (red), respectively [17]. The samples were examined by *z*-axis sectioning to allow a three-dimensional reconstruction of the skin.

##### Image Analysis

For each biopsy, three-dimensional images were obtained with Volocity software (Perkinmeyer). From these images, the fluorescence intensity (RFU.cm^2^) was quantified digitally at the gray level with the Image J software. The principle is based on pixel separation. The 16-bit image is separated into 256 gray levels (0 = black; 255 = white). The pixels of the areas containing the particles (light pixels) are quantified in relation to the total number of pixels in the selected image.

##### Immunohistochemistry

The frozen skin biopsies were cut using a cryomicrotome (Thermo Scientific, Illkirch-Graffenstaden, France) after being immersed in the cryomatrix at −50 °C. The chamber of the microtome was fixed at −20 °C and sections of 7 µm were prepared and used for the immunohistochemcial staining. The sections were fixed with 100% methanol for 5 min at −20 °C. Sections were then washed two times with phosphate-buffered saline containing 0.1% Tween 20 before being incubated with a 1% BSA solution in PBS. Samples were then washed and incubated with the primary antibody overnight at 4 °C and washed with the 0.1% Tween 20 in PBS solution. If needed, a secondary antibody was then added and incubated with the sections for 1 h at room temperature. Two steps of washing were then performed to remove the unreacted antibody; antibody dilutions were produced according to the manufacturer’s instructions. For the accurate identification of the epidermis layer, the nuclei of the cells were stained with DAPI for 10 min at room temperature before being washed and prepared for microscopical examination. The samples were examined using a confocal laser scanning microscope (CLSM) LSM 800 Zeiss (Zeiss, Marly Le Roi, France). For the determination of epidermal thickness, images were again analyzed using ImageJ software (National Institutes of Health, Bethesda, MA, USA). 

##### Statistical Analysis

All results are expressed as the mean and standard deviation. Statistical differences were calculated using the Chi-2 test or Fisher test for the qualitative variable between healthy volunteers and patients. The Chi2 Mac Nemar test or Student’s t test was used for the qualitative variables between the lesion and lesion-free skin of the patients. For semi-quantitative variables, the Wilcoxon or Kruskal–Wallis test was used. For each of the experiments, *p* values ≤ 0.05 were considered significant.

#### 2.2.4. Application of the Nanoparticles on Porcine Model of Atopic Dermatitis

For comparison purposes, a porcine atopic dermatitis model was employed and the same particles were applied to the healthy and inflamed parts of the skin, and the penetration profile was tracked by CLSM as described in Section Image Analysis. Female pigs of the Youna breed (average weight 35 kg, *n* = 3) were provided by the Gaec des 4 vents farm (Fontanès, France). A swine model of allergic dermatitis was developed by Dr. Pin’s team at VetAgro Sup (Lyon, France). Briefly, the sensitization phase included the topical application of 300 μL of a 3% oxazolone solution in acetone onto pigs’ ears on days 0, 2, and 4 after starting the experiment. On the 7th day, only 100 μL of the 3% oxazolone solution in acetone was applied to the ears. Two days later, 100 μL of 3% oxazolone in acetone was applied to the right flank of the animals on a previously stripped area of the skin. Twelve 6.15 cm^2^ circles were drawn to delineate the deposition areas. Two days after the induction of atopic dermatitis, 120 μL of the fluorescent nanoparticles were applied without a massage to the delineated sites on the right and left flanks. Then, 6 mm × 6 mm biopsies were taken from both the right and left flanks on day 1, day 3, and day 10 after nanoparticle application. The skin was then examined using CLSM as described in Section Image Analysis. The left flank was used as a control area with the same demarcations. The pigs were kept under pathogen-free conditions in approved facilities that complied with the standards imposed by the Ministry of Agriculture. At the end of the experiment, the pigs were euthanized. All procedures were conducted at the Claude Bourgelat Institute on the VetAgro Sup campus in Lyon and were approved by the Institute’s ethics committee.

## 3. Results and Discussion

The current work aimed at testing the penetration of polymeric nanoparticles into healthy human skin and comparing the penetration behavior to the lesional and non-lesional skin of atopic dermatitis patients. This was achieved via the preparation of fluorescent PLGA nanoparticles applied to different skin types and tracked by the CLSM. The use of immunohistochemical staining of the different proteins involved in the tight junctions that play an important role in the skin barrier properties and hence affect the nanoparticles’ penetration behavior then explained the results. Finally, the results were compared to the results obtained from the porcine dermatitis model to confirm the agreement between the clinical trial and animal results.

The fluorescent PLGA nanoparticles were successfully produced with a size of 139.4 ± 0.9 nm and a very homogenous particle size distribution, where the Polydispersity index was 0.038 ± 0.021. The zeta potential was slightly negative but almost neutral (−0.55 ± 0.35). The concentration of fluorescein sodium in the particles was found to be 2.6 ± 0.1 µg/mL. Regarding the pharmacopeial tests for the residual solvent and the microbial count, it was found that the residual solvent is 7 ppm, and microbial limits were confirmed. 

**Figure 1:**
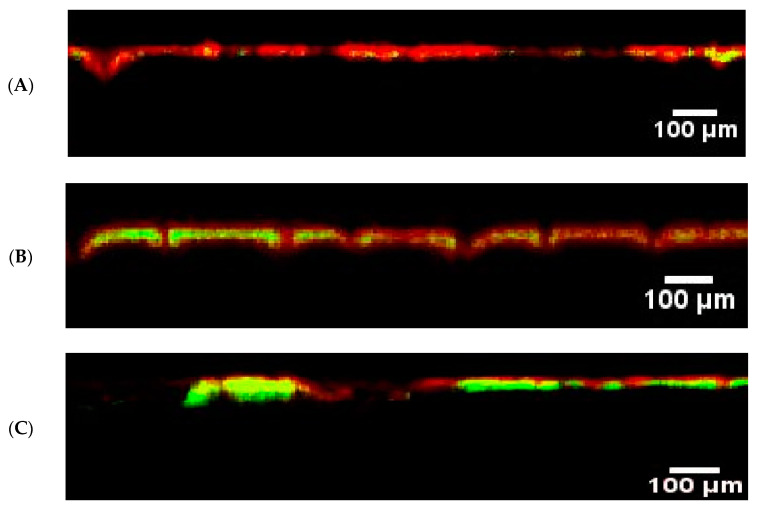
CLSM images of the penetration of the fluorescent PLGA nanoparticles (green fluorescence) into the skin surface (marked by the red auto-fluorescence) in (**A**): healthy skin, (**B**): non-lesional skin of AD patients, and (**C**): lesional skin of AD patient.

The penetration of fluorescent nanoparticles into the skin of healthy volunteers, non-lesional areas of AD patients, and lesions of AD was investigated using CLSM, as presented in Figure 1.

**Figure 2:**
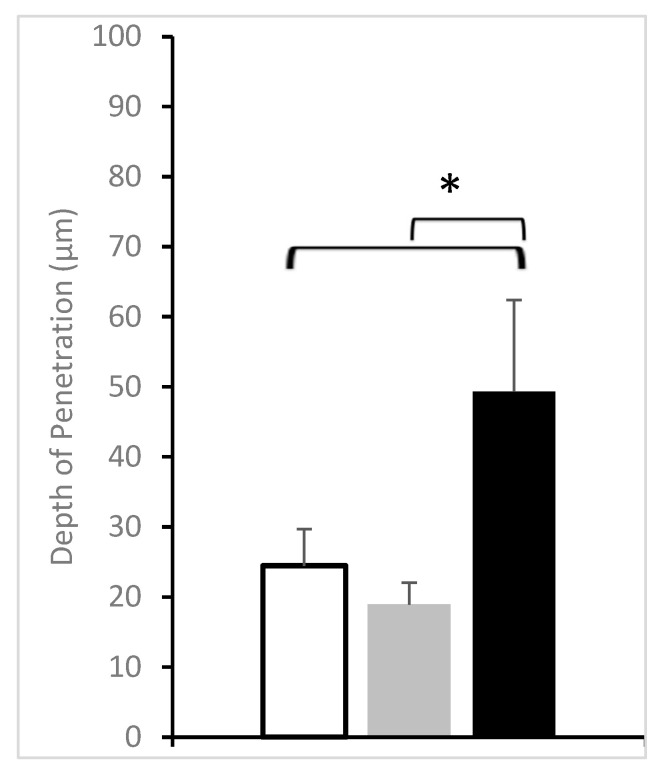
Depth of penetration of the nanoparticles on healthy volunteers’ skin (White bars), AD patients’ non-lesional skin (Grey bars), and AD patients’ lesional skin (Black bars). (*n* = 10 for healthy volunteers and *n* = 6 for AD patients). * represents significant differences.

**Figure 3:**
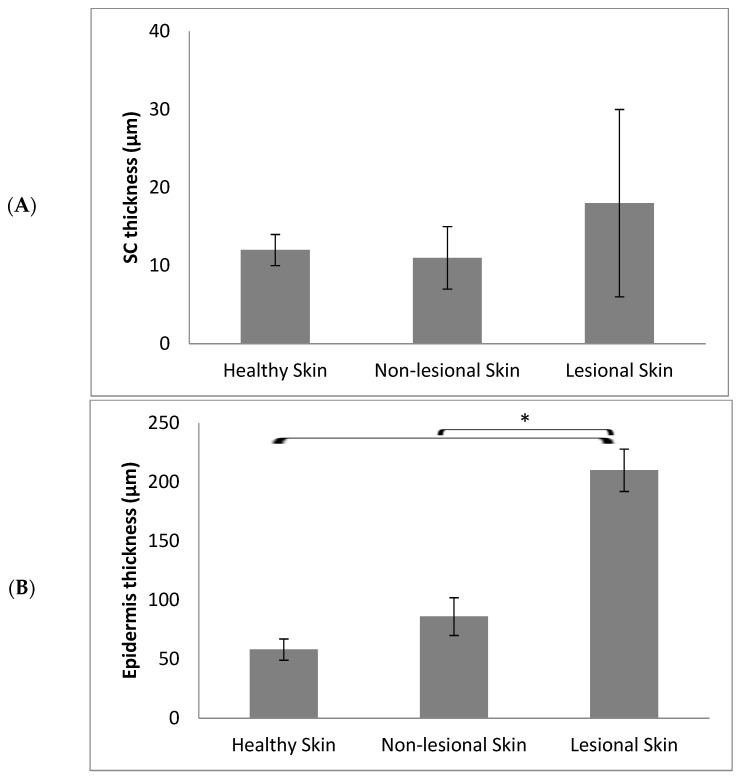
Average thickness of the stratum corneum (**A**) and epidermis layer (**B**) in the different types of skin. (*n* = 10 for healthy volunteers and *n* = 6 for AD patients). * represents significant differences.

As seen in the figure, in the case of the skin of healthy volunteers, the accumulation of the nanoparticles was minimal, and the signal of the green fluorescence was hardly detected (Figure 2). Similar results were obtained from the image analysis of nanoparticle penetration into the non-lesional parts of the AD patients. On the other side, a significantly higher accumulation was observed on the AD lesions. These observations are in line with previous studies performed on different animal models of dermatitis. There was a selective accumulation of polymeric nanoparticles of small size of around 100 nm in dithranol-induced dermatitis lesions in mice ears [11,12], as well as in oxazolone-induced dermatitis in mice and pigs [13]. Comparing the skin layer thicknesses, namely the stratum corneum and epidermis, in the different types of skin in the study revealed that the thickness of the SC was not significantly changed between healthy and AD skin, although was slightly higher in AD lesions. The healthy and non-lesional skin had comparable dimensions of 12 ± 2 and 11 ± 4 µm, while a higher value was observed for the lesional skin at 18 ± 12 µm. On the other side, there was a significant enlargement of the epidermal thickness in the case of AD lesions compared to healthy or non-lesional skin (Figure 3). This correlates well with epidermal hyperplasia, which is considered a histological hallmark in atopic dermatitis and plays a major role in the supply of proinflammatory mediators from keratinocytes [18]. It was found that the average epidermal thickness was 58 ± 9 µm in the healthy volunteers’ skin compared to 86 ± 16 µm and 210 ± 18 µm in the non-lesional and lesional skin of atopic dermatitis patients, respectively. This meant that the enhanced nanoparticle accumulation in the inflamed lesions was still limited to the upper layers of the epidermis, providing more proof that polymeric nanoparticles offer a potential carrier for targeted drug delivery to inflamed skin with minimal deeper penetration and a lower probability of systemic absorption [19]. 

The different penetration behavior of the fluorescent PLGA nanoparticles in the different skin types could be related to the altered barrier properties of the skin in each case. Although the most important player in the skin barrier properties is the stratum corneum, the tight junctions function as a gate for the passage of water or water-soluble molecules through the paracellular mechanism. Epidermal tight junctions interact in a dynamic way with the SC to form the intact barrier of the skin. Several types of transmembrane proteins are involved in the structure of the tight junctions such as Claudins, junctional adhesion molecules (JAM), occluding, tricellulin, and zonulae occludens (ZO-1, 2, and 3) [20]. Claudin-1, ZO-1, and claudin-4 have been identified to be among the most important proteins contributing to the barrier function of the skin. Therefore, they were selected to be studied in the current work to investigate their representation in the different skin types and their influence on the barrier properties and the subsequent nanoparticle penetration into the skin. 

**Figure 4:**
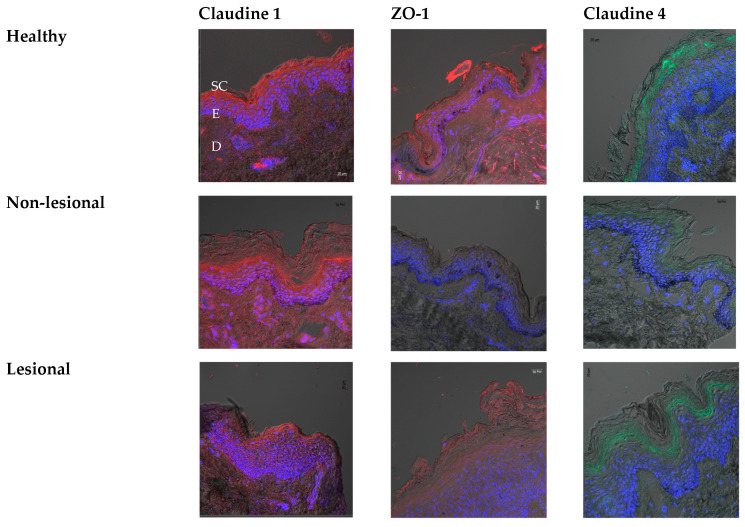
CLSM images showing the expression of the Claudin-1, ZO-1, and Claudin-4 in healthy volunteers and non-lesional and lesional skin of AD patients. Scale bars = 20 µm. The blue stain marks the nuclear staining of the epidermis layer (E), which lies in between the upper stratum corneum (SC) layer and the lower dermis layer (D).

Barrier properties of lesional AD skin were reported to be compromised as seen from the elevated Trans-epidermal water loss (TEWL) values compared to the non-lesional skin or healthy people’s skin [21]. Although the TEWL values of non-lesional skin tend to have relatively higher values compared to the control, the differences are usually not significant [22]. The intensity of the fluorescence obtained from the Claudin-1 immunostained sections has shown a similar expression in both healthy and non-lesional skin sections. However, it was found that the intensity was much less in the sections of AD lesions (Figure 4). This is in agreement with previous studies where there was neither a difference in Claudin-1 intensity in the upper (stratum granulosum and upper stratum spinosum) nor in the lower (stratum basale and lower layers of stratum spinosum) epidermal layers between non-lesional AD and controls [21]. However, reduced expression of Claudin-1 was reported in AD patients, which was not observed in psoriasis patients. This is expected to play a great role in the barrier properties of the skin where Claudin-1 represents an important component of the tight junctions in the granular layer of the epidermis [23]. The degree of downregulation of Claudin-1 in AD lesion skin was correlated to the dermal infiltration intensity, suggesting that the decreased mRNA expression of Claudin-1 is triggered by skin inflammation [24]. Claudin-1’s downregulation also correlated with the Th-2 inflammation pathway and was also aggravated by scratching of the skin [25].

Regarding Claudin-4, we observed decreased immune intensity in non-lesional skin compared to the healthy controls, while lesional skin has shown a similar intensity to healthy skin. Yet, we assume that due to the hyperplasia observed in the lesional skin, in total, the amount of protein decreased in relation to the skin tissue in the case of AD. Previously, it was reported that the downregulation of Claudin-1 in AD lesions might sometimes be accompanied by the increased production of Claudin-4 as a compensatory mechanism to restore the barrier properties and integrity of the skin [21]. However, this seems to be insufficient as seen from the higher deposition of the nanoparticles in the AD lesions. Decreased synthesis of Claudin-4 in non-lesional sites of AD patients’ skin has been previously detected by Western blot analysis, while comparable levels were obtained in AD lesions relative to the healthy control [26]. ZO-1 was found to be significantly reduced in both the lesional and non-lesional skin of AD patients, an observation that was confirmed in our study. These results suggest that the tight junctions’ function could also be impaired in the non-lesional parts of the skin. This was owing to the abnormal immunological abnormalities associated with AD. Exposing the keratinocytes to very low concentrations of IL-17 (1 ng/mL) led to a significant reduction of trans-epithelial electric resistance while no changes were observed with IL-4, IL-22, and TNF-alpha, thus embracing the major role of IL-17 on the impaired function of tight junctions [26]. 

**Figure 5:**
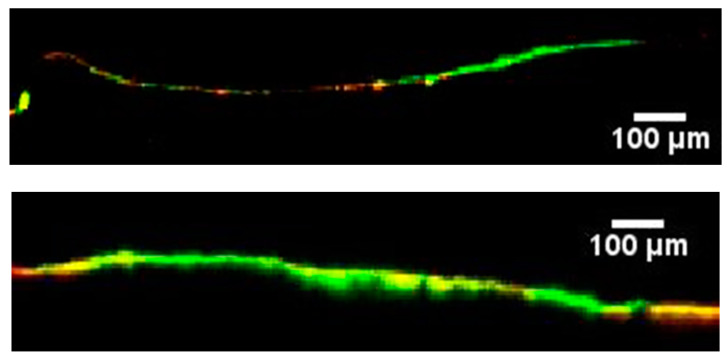
CLSM images showing the depth of penetration of nanoparticles into healthy porcine skin (**up**) versus inflamed porcine skin (**down**).

Regarding the penetration of the same nanoparticles into the healthy and inflamed skin of pigs, it was found that there was deeper selective penetration into the inflamed skin, while the barrier properties of the healthy skin prevented any deeper penetration (Figure 5). These observations confirmed the results obtained from the clinical study and are consistent with the preclinical study performed earlier in our laboratory [13]. In this previous study, PLGA covalently bound to fluorescein was used to prepare nanoparticles with two different sizes, 70 and 300 nm. The penetration of both sizes was investigated in healthy skin and on oxazolone-induced dermatitis skin. By analyzing the quantity of fluorescence and the depth of penetration of these particles into porcine-inflamed skin and comparing them to the results of penetration of particles in our current study (with a particle size of around 140 nm), it was found that the 140 nm particles had an intermediate penetration depth between both particles. This confirms the size dependence of the nanoparticles’ penetration into the inflamed skin [27].

## 4. Conclusions

The current work confirmed the great interest of polymeric vectors in dermatology thanks to their specificity of penetration in inflamed skin and their higher cutaneous deposition in atopic dermatitis lesions. The use of such vectors would allow better management of chronic inflammatory skin diseases while limiting the risks of local adverse effects. PLGA nanoparticles can be used for the topical drug delivery of various drugs and immunomodulators such as tacrolimus or cyclosporine with minimal side effects on the healthy parts of the skin or the systemic circulation. Several inflammatory skin diseases such as atopic dermatitis, psoriasis, or pemphigus could benefit from this targeted delivery system. 

## Figures and Tables

**Table 1:** Number and characteristics of the healthy volunteers and AD patients selected for the study.

	Sex (M/F)	Average Age (Years) Median [min–max]
Healthy volunteers	5/5	31 [21–42]
Atopic dermatitis Patients	2/4	26.5 [21–30]

## Data Availability

Data available on request.
